# Current Knowledge of the Molecular Pathogenesis of Cutaneous Lupus Erythematosus

**DOI:** 10.3390/jcm12030987

**Published:** 2023-01-27

**Authors:** Fumi Miyagawa

**Affiliations:** Department of Dermatology, Nara Medical University School of Medicine, 840 Shijo, Kashihara, Nara 634-8522, Japan; fumim@naramed-u.ac.jp

**Keywords:** cutaneous lupus erythematosus, type I interferon, plasmacytoid dendritic cells, Th1 cells, apoptosis, toll-like receptors, environmental factors, genetic factors

## Abstract

Cutaneous lupus erythematosus (CLE) is an autoimmune disease, which can be limited to the skin or associated with systemic lupus erythematosus (SLE). Gene expression analysis has revealed that both the innate and adaptive immune pathways are activated in CLE. Ultraviolet (UV) light, the predominant environmental factor associated with CLE, induces apoptosis in keratinocytes, and the endogenous nucleic acids released from the apoptotic cells are recognized via pattern recognition receptors, including Toll-like receptors. This leads to the production of type I interferon, a major contributor to the pathogenesis of CLE, by plasmacytoid dendritic cells. UV irradiation can also induce the externalization of autoantigens, such as SS-A/Ro, exposing them to circulating autoantibodies. T-helper 1 cells have been reported to play important roles in the adaptive immune response to CLE. Other environmental factors associated with CLE include drugs and cigarette smoke. Genetic factors also confer a predisposition to the development of CLE, and many susceptibility genes have been identified. Monogenetic forms of CLE also exist. This article aims to review current knowledge about the pathogenesis of CLE. A better understanding of the environmental, genetic, and immunoregulatory factors that drive CLE may provide important insights for the treatment of CLE.

## 1. Introduction

Cutaneous lupus erythematosus (CLE) is an autoimmune disease, which can present as a cutaneous manifestation within the spectrum of systemic lupus erythematosus (SLE) or as an isolated cutaneous lupus lesion without any evidence of SLE (discoid LE (DLE) or subacute CLE (SCLE)) [1,2]. Similar to SLE, the pathogenesis and clinical manifestations of CLE are considered to be complex and heterogeneous, and both genetic and environmental factors are involved in the development of CLE. However, the pathogenesis of CLE is less clear than that of SLE. Given that CLE has various subtypes, each subtype may have a unique pathophysiology [2].

This review provides an overview of the clinical features and current knowledge of the molecular pathogenesis of CLE, particularly its immunological, genetic, and environmental aspects.

## 2. Clinical Features of CLE

### 2.1. Classification

CLE has various subtypes, each of which may have a unique pathophysiology. CLE is classified into LE-specific skin lesions, which are characterized histopathologically by interface dermatitis, and LE-non-specific skin lesions, such as urticarial vasculitis and livedo reticularis [3]. LE-specific skin lesions can be subdivided into three major subtypes: chronic CLE (CCLE), SCLE, and acute CLE (ACLE) on the basis of their clinical, laboratory, and histological features and how long the skin lesions last (Table 1) [3,4,5]. Another more recently defined subtype has been termed LE tumidus (LET) and described as intermittent CLE (ICLE) [5,6]. The most common subtype of CLE is DLE, which accounts for 80% of cases [7].

**Figure 1:**
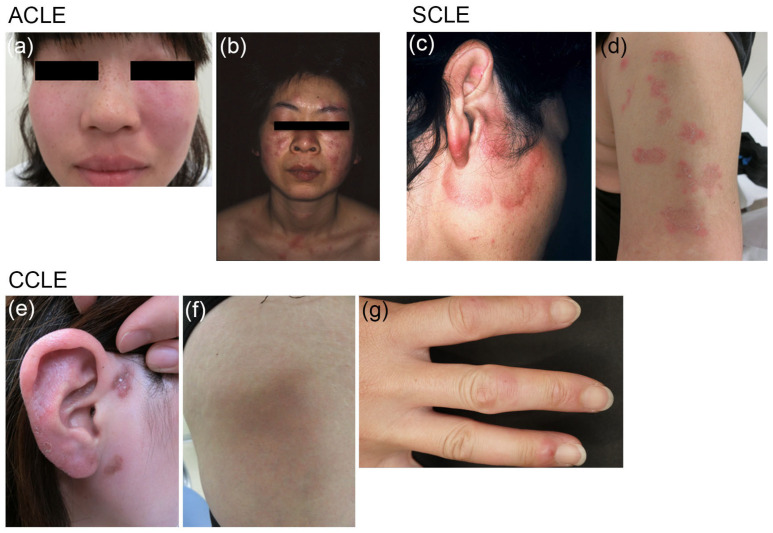
Typical clinical presentations of three subtypes of CLE. ACLE: (**a**) Malar rash or butterfly rash on the face; (**b**) Widespread indurated erythema on the face, neck, upper chest, and shoulders. SCLE: (**c**) Annular-polycyclic lesions on the face; (**d**) Papulosquamous or psoriasiform lesions on the upper arm. CCLE: (**e**) Scar-causing DLE lesions on the face; (**f**) Lupus erythematosus profundus on the thigh, showing depressions; (**g**) Chilblain lupus erythematosus on the fingers, resembling frostbite.

CCLE has multiple subtypes, including DLE, lupus erythematosus profundus, and chilblain lupus erythematosus (Figure 1), with the most frequent subtype being DLE [1,4]. DLE is characterized by well-demarcated, atrophic scarring, and hypopigmented plaques in the head and/or neck region, while SCLE usually manifests as widespread, non-scarring lesions with scaling, depigmentation, and telangiectasis on the light-exposed areas of the face, neck, upper trunk, upper back, shoulders, and arms. It typically presents as papulosquamous lesions and/or annular plaques [1,4] (Figure 1). ACLE produces indurated erythematous lesions on the malar areas of the face (a malar rash or butterfly rash), which typically cross both cheeks, but spare the nasolabial folds, and also cause widespread indurated erythema (on the face, scalp, neck, upper chest, shoulders, arms, and the backs of the hands) [3,8] (Figure 1). ACLE typically manifests concurrently with other symptoms of SLE.

### 2.2. Epidemiology

The age- and sex-adjusted incidence of CLE ranges from 4–4.3 per 100,000 [7,9,10], while that of SLE ranges from 2.78–2.9 per 100,000 [9,10]. A small percentage of CLE patients subsequently develop systemic manifestations. In a previous study, 24% of CLE patients had already been diagnosed with SLE at the time they were diagnosed with CLE, and an additional 18% were diagnosed with SLE within three years of being diagnosed with CLE, with the probability of SLE progression being highest for the patients with SCLE [7]. Another study showed that 12.2% of CLE patients underwent disease progression to SLE within four decades [10].

## 3. Pathogenesis of CLE

### 3.1. Gene Expression Patterns

Microarray-based gene expression analysis has revealed strong activation of both the innate and adaptive immune pathways in DLE skin lesions [11]. The innate immune pathways that exhibited upregulated expression included nucleic acid recognition mechanisms (the Toll-like receptor (TLR) signaling pathway, cytosolic DNA-sensing pathway, retinoic acid-inducible gene I [RIG-1]-like receptor signaling pathway, and the nucleotide-binding oligomerization domain [NOD]-like receptor signaling pathway), their downstream pathways (cytokine-cytokine receptor interactions, chemokine signaling pathways, and the Janus kinase-signal transducer and activator of transcription [JAK-STAT] signaling pathway), and apoptosis. The adaptive immune pathways that demonstrated upregulated expression included antigen processing and presentation, T-cell and B-cell receptor signaling pathways, and leukocyte transendothelial migration [11].

### 3.2. Autoantibodies

Autoantibodies may be related to the pathogenesis of CLE, since the deposition of immunoglobulins and complements along the dermal-epidermal junction has been demonstrated in LE patients, especially in sun-exposed skin [12]. Many CLE patients develop autoantibodies, including antinuclear antibodies (ANAs), although ANAs are only found in 10–30% of DLE patients and approximately 70% of SCLE patients, whereas they are found in over 90% of SLE patients. In particular, anti-SS-A/Ro and anti-SS-B/La antibodies are frequently detected in CLE patients [13]. Direct evidence to show an association between anti-SS-A/Ro antibodies and the pathogenesis of SCLE has been provided by Lee et al. [14]. They showed that the injection of anti-SS-A/Ro antibodies into human skin-grafted mice resulted in epidermal IgG deposition in a similar pattern to that seen in the lesions of SCLE patients and that preabsorbing anti-SS-A/Ro serum with Ro abolished binding to the human skin grafts [14]. Greiling et al. showed that commensal bacteria in the human skin, oral, and gut microbiotas contain Ro60 orthologs and that T cells and B cells from lupus patients with Ro60 autoantibodies responded to these commensal Ro60 orthologs in vitro. In vivo studies confirmed that the monocolonization of the mouse gut with Ro60 ortholog-producing commensal bacteria triggered lupus-like symptoms, including the production of anti-human Ro60 autoantibodies and the development of glomerular immune complex deposits, suggesting that commensal Ro60 ortholog cross-reactivity is involved in the pathogenesis of lupus [15].

### 3.3. Toll-Like Receptors

TLRs have been implicated in SLE, since they are involved in the activation of autoreactive B cells, plasmacytoid dendritic cells (pDCs), and other cell types implicated in the pathogenesis of SLE. Since antibodies to RNA- and DNA-containing autoantigens are characteristic features of SLE, TLR7 and TLR9 in particular, have been implicated in the pathogenesis of SLE because TLR7 and TLR9 recognize ssRNA and CpG DNA, respectively, and initiate type I interferon (IFN) production [16]. However, although they exhibit similar tissue expression and are involved in similar signaling pathways, TLR7 and TLR9 have opposing inflammatory and regulatory roles in lupus-prone MRL/lpr mice [17]. TLR9-deficient MRL/lpr mice develop more severe clinical disease, involving more activated lymphocytes and pDCs, increased serum IgG and IFN-α levels, and accelerated lupus nephritis and mortality, although their ability to produce antibodies to DNA autoantigens (nucleosomes) is impaired. In contrast, TLR7-deficient MRL/lpr mice exhibit less severe disease involving less lymphocyte activation, decreased serum IgG levels, impaired generation of antibodies to RNA autoantigens (Sm and RNP), and less severe lupus nephritis [17]. In parallel with the severity of their systemic manifestations, TLR9-deficient MRL/lpr mice showed exacerbated skin disease, while TLR7-deficient MRL/lpr mice developed very little skin disease [17]. These findings suggest the importance of TLR signaling in the pathogenesis of CLE.

### 3.4. Cytokines

#### 3.4.1. Type I IFNs

The central role of type I IFNs in the pathogenesis of SLE has been well described [18,19]. The type I IFN pathway is also considered to be the major pathway involved in the pathogenesis of CLE. Patients with SCLE and DLE exhibit increased expression of type I IFN-regulated genes or the IFN signature in their peripheral blood, regardless of the presence/absence of concomitant SLE, which is correlated with their cutaneous disease activity [20]. Increased expression of IFN-regulated genes has also been demonstrated in lesional skin; i.e., overexpression of the myxovirus resistance protein 1 (MxA), which is specifically induced by type I IFNs and is used as a surrogate marker of local type I IFN production, has been detected in CLE lesions, regardless of the CLE subtype [21,22,23]. An analysis of the transcriptome of DLE lesions demonstrated that IFN signaling was upregulated [24]. However, the mechanisms by which IFNs lead to CLE lesions are still poorly understood. Using RNA-seq- and bioinformatic-based approaches, Tsoi et al. demonstrated that keratinocytes derived from SLE patients exhibited different responses to IFN stimulation than those of healthy individuals; i.e., the lupus patients’ keratinocytes showed a significantly hypersensitive response to IFNs [25]. They also identified paired-like homeodomain 1 (PITX1) as an upstream regulator of these IFN responses [25].

The source of type I IFN in CLE was evaluated by Sarkar et al., who found that the expression of keratinocyte-derived IFN-k, another type I IFN, was significantly higher in CLE lesions than in healthy skin [26]. IFN-k is responsible for maintaining baseline type I IFN responses in healthy skin; however, in CLE increased levels of IFN-k contribute to amplifying and accelerating the responsiveness of epithelia to IFN-α and increasing keratinocyte sensitivity to UV radiation [26].

Consistent with the critical role of type I IFNs in the pathogenesis of both SLE and CLE, blocking type I IFN signaling was shown to be effective against not only SLE, but also against skin lesions in SLE patients [27]. Anifrolumab, which has been approved for the treatment of moderate-to-severe SLE, is a fully human IgG1κ monoclonal antibody, which binds to the type I IFN-α receptor subunit 1 and inhibits signaling by all type I IFNs [27]. Further studies revealed that anifrolumab treatment resulted in greater improvements in the mucocutaneous system in SLE patients [28] and refractory CLE [29], suggesting that type I IFNs are central pathogenic mediators in CLE.

#### 3.4.2. Interleukin-6

Interleukin (IL)-6 has also been implicated in the pathogenesis of CLE. Mice with a Jak1 (p.Ser645Pro) point mutation, which induced constitutive activation of the JAK/STAT pathway, exhibited CLE-like skin lesions [30]. Molecular analysis of the skin lesions of the *Jak1*^S645P+/−^ mice revealed increased levels of IL-6 and phosphorylation of Stat3, which were indicative of activation of the IL-6-JAK-STAT pathway, in CLE [30]. Biopsy samples from DLE and SCLE skin lesions also showed significantly upregulated IL-6 expression, and keratinocytes from the unaffected skin of lupus patients produced significantly more IL-6 than those from healthy control subjects after treatment with TLR2, 3, or 4 agonists or UVB radiation [31]. This IL-6 hyperproduction was type I IFN-dependent, as pretreating keratinocytes with type I IFN increased their IL-6 production, and type I IFN blockade decreased IL-6 secretion by the lupus patients’ keratinocytes [31].

### 3.5. Apoptosis

In SLE, increased apoptosis due to the dysregulation of apoptosis and altered clearance of apoptotic cells leads to the augmented release of nuclear autoantigens, which promotes autoantibody production [32,33,34]. The subsequent formation of immune complexes of autoantibodies and nuclear autoantigens stimulates the production of IFN-α from pDCs via TLRs, particularly TLR7 and TLR9 [35]. These mechanisms, however, cannot be simply applied to CLE because autoantibodies are found infrequently in CLE patients [13], especially in DLE, the most common subtype of CLE, suggesting that different mechanisms drive the lesional activation of keratinocytes in CLE patients [6]. Kuhn et al. examined 85 skin biopsy specimens from patients with various subtypes of CLE using in situ nick translation and TUNEL to detect apoptosis. They found a significantly increased number of apoptotic cells in the CLE lesions [36]. Furthermore, apoptotic cells accumulate in the skin of CLE patients after UV irradiation, probably as a result of impaired or delayed clearance [36]. UV irradiation-induced keratinocyte apoptosis results in the release of endogenous nucleic acids (RNA and DNA motifs) and induces IFN-associated responses in cultured keratinocytes via pathogen recognition receptors [11]. The fact that three-prime repair exonuclease 1 (TREX1)-knockout mice lacking TREX1 exonuclease, which is involved in the clearance of cytosolic DNA motifs, developed CLE lesions when irradiated by UV light confirmed this hypothesis [11]. mRNA sequencing analysis of skin biopsies obtained from both the photoprovoked and unexposed skin of the same individual revealed that UV irradiation upregulated the expression of IFN-regulated genes and the major histocompatibility complex (MHC) II gene in CLE skin, but not in healthy skin [37].

The MRL/lpr mouse, an animal model of human SLE, spontaneously develops skin lesions similar to those found in human CLE, beginning at the age of 3 months [38,39]. These mice are characterized by the *lpr* mutation, which is a defect in the Fas antigen. MRL/n mice, the control counterpart of MRL/lpr mice lacking the *lpr* mutation, develop less severe CLE lesions than MRL/lpr mice as they age. These results suggest that a defect in the Fas antigen, which has been reported to mediate apoptosis, accelerates the progression of mild forms of the systemic and cutaneous manifestations into more severe forms in MRL mouse strains [39]. The critical role of the Fas ligand (FasL) in the formation of CLE has also been demonstrated by Mande et al. [40]. They developed an inducible model of systemic autoimmunity that depends on the adoptive transfer of ovalbumin (OVA)-specific DO11 T cells into sublethally irradiated TLR9^KO^ mice expressing a transgenically encoded OVA fusion protein on MHC class II^+^ cells. These mice only developed severe CLE lesions if the recipient was TLR7-sufficient, and DO11 T cells were capable of expressing FasL, as the adoptive transfer of FasL-deficient DO11^gld^ T cells completely failed to elicit overt CLE lesions [40]. Consistent with these murine models, FasL expression was found to be significantly increased in all SCLE and CCLE patients, but not in psoriasis patients, verifying that its expression is specific to lupus rather than all inflammatory skin diseases [40].

Photosensitivity, one of the major symptoms of SLE, has been proposed to occur as a consequence of antibody-dependent keratinocyte damage following the binding of antibodies to SS-A/Ro, SS-B/La, and U1 ribonucleoprotein (U1RNP) antigens expressed on the surfaces of UV-irradiated human keratinocytes [41]. A link between apoptosis and the development of autoantibodies has been shown by Casciola-Rosen et al., who demonstrated that during UVB-induced keratinocyte apoptosis, subcellular autoantigens, such as Ro, La, and U1-RNP, were translocated into two distinct blebs on the cell surface [42]. In vitro studies have shown that autoantigens that are often recognized by the autoantibodies produced by patients with LE are clustered in the surface blebs of apoptotic keratinocytes [42].

### 3.6. Immune Cells

#### 3.6.1. Plasmacytoid Dendritic Cells

Immunofluorescent staining of skin samples from DLE and SLE patients revealed that the CLE lesions had been infiltrated by large numbers of pDCs, the principal natural IFN-producing cell, and the density of pDCs correlated well with the high number of cells expressing the IFN-α/β-inducible protein MxA, suggesting that pDCs produce IFN-α/β locally [43].

Guiducci et al. developed a mouse model of CLE by subjecting (NZBxNZW)F1 mice to tape stripping. In this model, the depletion of pDCs or treatment with a bifunctional TLR7/9 inhibitor prevented the development of CLE lesions, suggesting that CLE lesion formation is dependent on pDC activation by nucleic acids via TLR7 and TLR9 [44].

#### 3.6.2. T-Helper 1 Cells

CLE is considered to be a T-helper 1 (Th1)-dominated disease. Transcriptional analyses of DLE skin showed the activation of the IFN-γ pathway as well as type I IFNs, with a relatively minimal Th17 signature [24]. The extraction of T cells from DLE skin lesions revealed the predominance of IFN-γ-producing Th1 cells and the absence of IL-17-producing Th17 cells [24]. In addition, transcriptomic analysis of whole skin and analyses of infiltrating T cells confirmed that DLE is skewed toward the Th1 signature. The Th1-biased inflammatory immune response was shown to be induced by the local production of type I IFNs in CLE via the induction of IFN-inducible chemokines, such as IFN inducible protein 10 (IP10)/C-X-C motif chemokine ligand 10 (CXCL10), leading to the recruitment of CXC receptor 3 (CXCR3)-expressing T cells into skin lesions [45].

### 3.7. Environmental Factors

#### 3.7.1. Ultraviolet

UV light is one of the major provoking factors for CLE. Foering et al. investigated self-reported photosensitivity in 91 CLE patients and found that 81% of CLE patients had photosensitive skin lesions [46]. In a prospective, cross-sectional, multicenter study of 1002 CLE patients from the European Society of Cutaneous Lupus Erythematosus (EUSCLE), a history of photosensitivity was seen in 82.2% of ACLE patients, 73.7% of SCLE patients, 59.9% of CCLE patients, and 75.4% of ICLE patients [47]. However, the exact mechanism by which UV irradiation induces CLE remains unknown. In vitro experiments using keratinocytes obtained from the normal unaffected skin of 29 CLE patients showed that the keratinocytes from SLE and SCLE patients were more sensitive to UV radiation than healthy keratinocytes and showed enhanced susceptibility to antibody-dependent cell-mediated cytotoxicity (ADCC) by autologous patient’s serum or anti-SS-A/Ro^+^ serum [48].

Gehrke et al. proposed that UV irradiation-induced enhanced immune sensing by oxidized self-DNA may be involved in the UV light hypersensitivity seen in CLE. Immunobiological analysis of the skin samples of patients with UV-light-induced LE lesions revealed the colocalization of the oxidized base 8-hydroxyguanosine (8-OHG), a marker of oxidative damage in DNA, and the type I IFN-induced gene MxA. They subsequently demonstrated that the ears of MRL/lpr mice that had been repeatedly injected with UV-damaged oxidized DNA developed skin lesions, confirming that oxidation damage to self-DNA was able to trigger lupus lesions. Furthermore, they found that oxidized DNA confers resistance to cytosolic nuclease TREX-1 degradation, resulting in the stimulator of interferon response cGAMP interactor 1 (STING)-dependent recognition of oxidized DNA [49]. Inversely, bone marrow-derived DCs from TREX1-deficient mice produced high amounts of type I IFN in response to cytosolic DNAs irrespective of whether these DNAs were unmodified or oxidized, and TREX1-deficient mice developed skin lesions when injected with cytosolic DNAs [49]. These findings suggest that structural changes in DNA and cellular stress due to TREX1 mutations increase UV-induced DNA damage, thereby enhancing inflammation and type I IFN induction, which may contribute to the induction of CLE lesions in predisposed individuals.

#### 3.7.2. Cigarette Smoke

Smoking is one of the environmental factors associated with CLE. A prospective cohort study showed that current smokers with CLE had worse disease with higher Cutaneous Lupus Erythematosus Disease Area and Severity Index (CLASI) scores, had a worse quality of life, and were treated more often with a combination of hydroxychloroquine and quinacrine than non-smokers [50].

#### 3.7.3. Drugs

So far, over 100 drugs have been reported to induce CLE or CLE flares [51,52]. Unlike idiopathic CLE, in which DLE is the most common subtype, SCLE is the most frequently described form of drug-induced CLE [51,52]. Numerous drugs have been implicated in the induction of drug-induced SCLE (DI-SCLE) (Table 2) [53,54]. The most common drugs involved used to be antihypertensives, particularly thiazide diuretics and calcium channel blockers, followed by antifungals, particularly allylamine antifungals [53]. Recently, new medications, such as biologics, particularly anti-tumor necrosis factor (TNF)-α agents, proton pump inhibitors, and chemotherapeutics, have become more common causes of DI-SCLE [51,55].

In rare cases, a discoid form of CCLE (DI-CCLE) can be caused by medication [51,52] (Table 3). DI-CCLE has mostly been reported to be associated with 5-fluorouracil (FU), non-steroidal anti-inflammatory drugs (NSAIDs), and anti-TNF-α agents [51,52].

Since the causal relationship between drugs and LE is clear, a mouse model of drug-induced LE involving treatment with 5-FU and UVB has been established, although 5-FU alone did not induce marked clinical and histological changes [56]. In this model, DLE-like skin lesions were induced in C57BL/6 J (B6) mice treated with a high dose of 5-FU (2 mg) plus UVB. Interestingly, TCR-α^−/−^ mice developed more severe skin lesions than B6 mice, even after the administration of a low dose of 5-FU (0.2 mg) plus UVB, suggesting that αβ T cells are required to protect against the development of drug-induced DLE [56].

### 3.8. Genetic Factors

#### 3.8.1. Mutations

Familial chilblain lupus (FCL) is a well-known monogenetic form of CLE, which is inherited as an autosomal dominant trait and characterized by cold-induced erythematous skin lesions, which arise in acral locations and first occur in early childhood. FCL has been reported to be caused by heterozygous loss-of-function mutations in the *TREX1* gene, which encodes 3′–5′ repair exonuclease 1 [57,58], or the *SAMHD1* (sterile alpha motif domain and HD domain-containing protein 1) gene, which encodes deoxynucleotide-degrading phosphohydrolase [59]. The identified mutation in *TREX1* caused reduced exonucleolytic activity [57,58], which can lead to the impairment of granzyme A-mediated caspase-independent apoptosis [58]. FCL patients with *TREX1* mutations as well as *TREX1*-deficient mice have been shown to exhibit type I IFN activation [60] due to uncontrolled accumulation of cytoplasmic self-DNA, representing a danger signal activating the innate immune system [61,62]. A recent study demonstrated that a heterozygous gain-of-function mutation in *STING* also causes FCL by inducing constitutive type I IFN activation [63]. The abovementioned genes implicated in FCL suggest that FCL has genetic causes relating to components of the type I IFN signaling pathway.

Congenital deficiencies in early components of the complement system, such as C1q, C1r, C1s, C2, or C4, are known to be associated with the development of SLE. Complement deficiencies, such as deficiencies of C2 and C4, have also been reported to be associated with the development of CLE [64,65,66].

#### 3.8.2. Polymorphisms

Jarvinen et al. investigated whether single-nucleotide polymorphisms (SNPs), which have previously been shown to increase the risk of SLE, were also associated with CLE in Finnish patients with CLE (177 DLE patients and 42 SCLE patients) [67]. The known SLE susceptibility genes selected for genotyping included Fcγ receptor 2A (*FCGR2A*), cytotoxic T-lymphocyte-associated protein 4 (*CTLA4*), programmed cell death 1 (*PDCD1*), interferon regulatory factor 5 (*IRF5*), GTPase, IMAP family member 5 (*GIMAP5*), and tyrosine kinase 2 (*TYK2*). Among them, *TYK2*, *IRF5*, and *CTLA4* showed associations with DLE, and *IRF5* was also found to be associated with SCLE [67]. These findings suggest that susceptibility genes that are strongly associated with SLE are also predisposing factors for CLE and that the type I IFN pathway contributes to the pathogenesis of CLE, as *TYK2* and *IRF5* are involved in the type I IFN pathway. Similarly, Skonieczna et al. investigated whether three SNPs that have been found to be associated with SLE were also associated with DLE in a Polish population [68]. They analyzed three SNPs located in the *STAT4*, integrin subunit alpha M (*ITGAM*), and tenascin XB (*TNXB*) genes, and found an association between *STAT4* polymorphism and the development of DLE. This indicates that differences exist between the molecular backgrounds of DLE and SLE, but the dysregulation of the type I IFN pathway seen in these conditions may have a common molecular background [68]. Another study demonstrated a significant association between SCLE and the *TNF-308A* allele, which has also been shown to contribute to susceptibility to SLE [69]. A SNP of the complement *C1QA* gene has been reported to be strongly associated with SCLE [70]. In addition, polymorphisms of the *ITGAM* gene, a susceptibility gene for SLE, result in a higher risk of DLE than SLE [71].

A large comprehensive genome-wide association study of 183 CLE patients (CCLE: 44.8%, SCLE: 40.4%, LET: 14.2%) identified candidate genes and genomic regions that may contribute to pathogenic mechanisms in CLE. These included human leukocyte antigen (*HLA*)-*DQA1*, casein kinase II subunit beta (*CSNK2B*), MutS protein homolog 5 (*MSH5*), MHC class I polypeptide-related sequence A (*MICA*), *HLA-DRB3*, *HLA-DRA*, mucin 21, cell surface associated (*MUC21*), *MICB*, flotillin 1 (*FLOT1*), tripartite motif-containing 39 (*TRIM39*)/Ribonuclease P/MRP subunit P21 (*RPP21*), psoriasis susceptibility 1 candidate 1 (*PSORS1C1*), and MAS1 proto-oncogene-like, G protein-coupled receptor (*MAS1L*) [5]. Most of these genes are involved in the type I IFN pathway (*TRIM39*/*RPP21*), apoptosis regulation (*MICA*, *MICB*, *TRIM39*/*RPP21*), or antigen presentation (*HLA-DQA1*) [1,5]. Some of these CLE-associated genes are also associated with SLE, suggesting that different, but partially overlapping, genes underlie CLE and SLE. These findings suggest that CLE and SLE are different diseases with partially overlapping clinical features [5].

### 3.9. Sex Bias

As with SLE, which occurs at a female:male ratio of 9:1–10:1, CLE also shows female skewing, which occurs at a female:male ratio of 3:1–4:1 [7,9,72]. Liang et al. demonstrated that a transcription factor, vestigial-like family member 3 (VGLL3), exhibited markedly female-biased expression in normal skin, and its expression was further elevated in CLE lesions in both males and females; they further demonstrated that VGLL3 is a critical regulator of the female-biased inflammatory genes associated with multiple autoimmune diseases, including B cell-activating factor (*BAFF)* and *ITGAM*, independent of sex hormone levels [73]. Transgenic mice that overexpressed VGLL3 in the epidermis exhibited a severe DLE-like skin rash, autoantibody production, and immune complex deposition in the skin and kidneys [74]. These results implicate VGLL3 as a master orchestrator of sex-biased autoimmunity, and the VGLL3-regulated gene network further promotes sex-biased autoimmunity [73,74].

## 4. Conclusions

CLE is a heterogeneous disease, and its pathogenesis has not been fully characterized. Interactions among multiple factors, including genetic factors; UV radiation; abnormalities in immune cells, such as T cells and DCs; inflammatory cytokines; and apoptosis, are likely to be involved in the pathogenesis of CLE. CLE, such as DLE, can be highly burdensome and in some cases can be resistant to therapies. A deeper understanding of the pathophysiology of CLE, particularly its molecular mechanisms and the dominant pathways active in CLE, may lead to the development of targeted and effective therapies. To this end, further research into this disease is needed.

## Figures and Tables

**Table 1:** Subtypes of CLE.

Subtype	Variant
ACLE	Localized (Malar rash) Generalized
SCLE	Annular Papulosquamous
ICLE	Lupus erythematosus tumidus
CCLE	Discoid lupus erythematosus (DLE) Localized Generalized Lupus erythematosus profundus Chilblain lupus erythematosus Hypertrophic CCLE Mucocutaneous CCLE

**Table 2:** Drugs implicated in the induction of DI-SCLE [53,54].

Class	Sub-Class	Drug
Antihypertensives	Thiazide diuretics	Hydrochlorothiazide, Hydrochlorothiazide + triamterene, Chlorothiazide
	Calcium channel blockers	Diltiazem, Verapamil, Nifedipine, Nitrendipine
	ACE inhibitors	Captopril, Cilazapril, Enalapril, Lisinopril, Ramipril
	Beta blockers	Acebutolol, Oxprenolol
Proton pump inhibitors		Lansoprazole, Esomeprazole, Omeprazole, Pantoprazole
Antifungals		Terbinafine, Griseofulvin
Antiepileptics		Carbamazepine, Phenytoin
Statins		Pravastatin, Simvastatin
Antihistamines		Ranitidine, Brompheniramine, Cinnarizine+thiethylperazine
Antibiotics		Amoxicillin+clavulanic acid, Ciprofloxacin
NSAIDs		Naproxen, Piroxicam
Chemotherapeutics		Docetaxel, Paclitaxel, Tamoxifen, Capecitabine, Doxorubicin, Gemcitabine, Masitinib, Mitotane, Palbociclib, Uracil-tegafur,
		5-Fluorouracil, Nivolumab, Pembrolizumab
Biologics	Anti-TNF	Etanercept, Infliximab, Adalimumab, Golimumab, Abatacept
	Anti-CD11a	Efalizumab
	Anti-IL-12/23	Ustekinumab
	Anti-IL-17	Secukinumab
Antidepressants		Bupropion
Immunomodulators		Leflunomide, IFN-α and β
Hormone-altering drugs		Leuprorelin, Anastrozole
Others		Allopurinol, Ticlopidine, Tiotropium, IVIG

**Table 3:** Drugs associated with DI-CCLE.

5-Fluorouracil
NSAIDs
TNF-α inhibitors
IVIG

## Data Availability

Not applicable.
